# Nickel Release, ROS Generation and Toxicity of Ni and NiO Micro- and Nanoparticles

**DOI:** 10.1371/journal.pone.0159684

**Published:** 2016-07-19

**Authors:** Siiri Latvala, Jonas Hedberg, Sebastiano Di Bucchianico, Lennart Möller, Inger Odnevall Wallinder, Karine Elihn, Hanna L. Karlsson

**Affiliations:** 1 Department of Environmental Science and Analytical Chemistry, Stockholm University, Stockholm, Sweden; 2 KTH Royal Institute of Technology, Division of Surface and Corrosion Science, School of Chemical Science and Engineering, Stockholm, Sweden; 3 Unit of Biochemical Toxicology, Institute of Environmental Medicine, Karolinska Institutet, Stockholm, Sweden; 4 Analytical Toxicology, Department of Biosciences and Nutrition, Karolinska Institutet, Huddinge, Sweden; VIT University, INDIA

## Abstract

Occupational exposure to airborne nickel is associated with an elevated risk for respiratory tract diseases including lung cancer. Therefore, the increased production of Ni-containing nanoparticles necessitates a thorough assessment of their physical, chemical, as well as toxicological properties. The aim of this study was to investigate and compare the characteristics of nickel metal (Ni) and nickel oxide (NiO) particles with a focus on Ni release, reactive oxygen species (ROS) generation, cellular uptake, cytotoxicity and genotoxicity. Four Ni-containing particles of both nano-size (Ni-n and NiO-n) and micron-size (Ni-m1 and Ni-m2) were tested. The released amount of Ni in solution was notably higher in artificial lysosomal fluid (e.g. 80–100 wt% for metallic Ni) than in cell medium after 24h (ca. 1–3 wt% for all particles). Each of the particles was taken up by the cells within 4 h and they remained in the cells to a high extent after 24 h post-incubation. Thus, the high dissolution in ALF appeared not to reflect the particle dissolution in the cells. Ni-m1 showed the most pronounced effect on cell viability after 48 h (alamar blue assay) whereas all particles showed increased cytotoxicity in the highest doses (20–40 μg cm^2^) when assessed by colony forming efficiency (CFE). Interestingly an increased CFE, suggesting higher proliferation, was observed for all particles in low doses (0.1 or 1 μg cm^-2^). Ni-m1 and NiO-n were the most potent in causing acellular ROS and DNA damage. However, no intracellular ROS was detected for any of the particles. Taken together, micron-sized Ni (Ni-m1) was more reactive and toxic compared to the nano-sized Ni. Furthermore, this study underlines that the low dose effect in terms of increased proliferation observed for all particles should be further investigated in future studies.

## Introduction

Human exposure to Ni in occupational settings is associated with a variety of pathological effects including skin allergies, lung fibrosis, and cancer of the respiratory tract [1,2]. Several Ni compounds such as high temperature green Ni oxide are classified as “human carcinogen via inhalation exposure” (Group 1Ai) [3], whereas Ni metal particles are classified as “possibly carcinogenic” (Group 2B) [4]. Pulmonary exposure to Ni-containing dusts and fumes is mostly common in metal refining and processing industries. However, the expanding production of Ni-containing nanomaterial presents an emerging concern [5]. Despite numerous studies on the toxicity of Ni, there is a lack of knowledge both on the characteristics and the effects of nano-sized Ni-containing particles.

Evidently, the ability of Ni-containing particles to release Ni is a crucial parameter from the risk assessment perspective. Skin irritation induced by Ni, for example, seems to be solely related to the released Ni species. There is also a known relationship between Ni release and skin sensitization [6]. Furthermore, according to the “Ni ion bioavailability”–model [7], the carcinogenic potential of Ni depends on the availability of Ni ions in the cell nucleus. This, in turn depends on the cellular uptake, intracellular Ni release, chemical speciation of released Ni, and on the transport of Ni into the nucleus. Although animal inhalation studies have shown that a ‘water-soluble’ Ni compound (Ni sulfate hexahydrate) is the most potent form of Ni to induce lung toxicity and possibly fibrosis [3], the same has not been shown for carcinogenicity. This is most likely due to the inefficient cellular uptake of extracellular Ni ions in combination with a rapid lung clearance of the water-soluble Ni species. Conversely, intracellular released Ni species have been linked to numerous mechanisms that are believed to be important for the carcinogenic potential of Ni compounds. Examples include the activation of stress-inducible and calcium-dependent signaling cascades, interference with DNA repair pathways [8] and epigenetic changes [9–11]. Most likely, the generation of reactive oxygen species (ROS) has a critical role in many of the observed effects. For example, ROS can cause various cell injuries including DNA damage or inhibition of DNA repair, which can lead to the preservation of DNA damage [12,13]. Nano-sized Ni and NiO particles have shown ROS generation in different model systems *in vitro* [14,15]. Furthermore, ROS has been suggested as an underlying reason for proliferative effects observed in human leukemia cells (X-CGD) at low Ni concentrations [16].

At present, only a very limited number of studies have investigated and compared Ni release from different Ni-containing particles [17,18]. Furthermore, comparative studies with a focus on micron- *vs*. nano-sized particles in combination with toxicological assessments are particularly rare. One of the few examples is presented by Pietruska and co-workers [19], who studied Ni release in cell medium as well as toxicity of NiO nanoparticles and Ni micro- and nanoparticles. It was shown that the nano-sized Ni particles released more Ni into the cell medium than the micron-sized Ni particles. Furthermore, the nano-sized Ni particles were also able to activate HIF-1α, which is a signaling pathway commonly activated by carcinogenic Ni compounds [19]. Similarly, Horie and co-workers [20] showed that nano-sized NiO particles exhibited both higher Ni release in cell medium and higher cytotoxicity when compared to micron-sized particles.

The aim of this study was to investigate and compare the characteristics of nickel metal (Ni) and nickel oxide (NiO) particles with a focus on Ni release and ROS generation, cellular uptake, cytotoxicity and genotoxicity. This was done by investigating the kinetics of Ni release, not only in cell medium but also in artificial lysosomal fluid (ALF). Ni release was also studied qualitatively inside the cells using TEM-imaging. Oxidative reactivity was assessed both by measuring acellular and intracellular ROS generation. A human type II alveolar epithelial cell line (A549) was chosen as the toxicological model, because the alveolar region is a likely deposition site for nano-sized, but also for some micron-sized particles. Furthermore, this cell line has previously been used in toxicological studies of metal and metal oxide particles [21,22].

## Materials and Methods

### Ni and NiO particles

Four different Ni and NiO particles were investigated in this study. Nano-sized Ni metal particle powder, here denoted “Ni-n” (<100 nm diameter, purity >99%, Cat#: 577995-5G, 93397KJ), and nano-sized Ni oxide particle powder, “NiO-n” (<50 nm diameter, >99.8% purity, Cat# 637130-25G, 17198PJ), were purchased from Sigma-Aldrich. Micron-sized Ni metal particle powders (“Ni-m1” and “Ni-m2”) were available in-house with further information about their identity provided in a previous publication by Mazinanian and co-workers [18]. The purity of Ni-m1 and Ni-m2 was 99.9% [18]. None of the particles were modified with surfactants or coatings. Originally, a micron-sized NiO particle powder was included in the study, but due to insufficient dispersion, this particle type had to be omitted (S1 Fig).

Particle suspensions were prepared from the powders freshly before each experiment. The powders were dispersed in artificial lysosomal fluid (ALF; pH 4.5; S1 Table), cell medium (supplemented Dulbeccos´s Minimal Essential Medium (DMEM^+^); pH 7.4) or phosphate buffered saline (PBS; pH 7.4) at a total Ni concentration of 1 mg mL^-1^. Particles were dispersed using a vortex mixer, after which they were sonicated in a water bath (30 min, 20°C, Elma, Transsonic Digital, at power-level 5). Suspensions were further diluted to reach the desired concentration for each investigation.

### Particle morphology and size

Transmission Electron Microscopy (TEM) investigations of the particle morphologies were performed using a Hitachi HT7700 microscope, operating at 100 kV. The samples were prepared by dispersing the particles in butyl alcohol at a concentration of 1 g L^-1^, after which the samples were sonicated (Branson Sonifier 250, 30% duty cycle, output 4, for 3 min). The suspensions were then pipetted onto TEM copper grids coated with carbon films (Ted Pella), from which the solvent evaporated at ambient laboratory conditions (25°C). TEM images were recorded in bright field mode.

Size measurements of the nano-sized particles were performed by means of photon cross-correlation spectroscopy (PCCS) using a Nanophox instrument (Sympatec GmbH, Germany). Prior to analysis, standard latex particles, sized 20 ± 2 nm (Nanosphere, Thermo Scientific), and blank samples (no added particles) were analyzed to ensure the accuracy of the measurements (data not shown). Size measurements of the micron-sized particles were performed with correlation spectroscopy by employing Malvern Mastersizer 2000 equipment with a Hydro SM dispersion unit. The particle suspensions were prepared by adding 1 mg of particles to 10 mL of cell medium (DMEM^+^), followed by sonication (as above). The sample volume was 1 mL, and the measurements were conducted at 20 ± 2°C in Eppendorf cuvettes (Eppendorf AG, Germany, UVette Routine pack, LOT no. C153896Q). A non-negative least square (NNLS) with a robust filter was used to determine particle size distributions from the correlation functions.

The BET-analysis (Brunauer–Emmet–Teller method) was performed in order to determine the specific surface area per mass (m^2^ g^-1^) of the particles [23]. A Micromeritics Gemini V instrument was used for the adsorption of nitrogen at cryogenic conditions, and for the measurements at five different partial pressures (p/p_0_ 0.10–0.25). The cross-sectional diameter of nitrogen (0.162 nm^2^) was used as an input parameter. The powders were dried in a tube by flushing with nitrogen for 30 min at 150°C. The measured mass was adjusted to correspond to an approximate total particle surface area of 1 m^2^.

### Ni concentration determination

Total Ni concentrations in the Ni release and cell-association experiments, as well as in the prepared particle suspensions, were determined by means of Atomic Absorption Spectroscopy (AAS). A digestion procedure was performed to ensure that Ni concentrations could be accurately quantified (acceptable recovery for added Ni particles, 85–100%). The samples (2.5 mL) were mixed with 1 mL H_2_O_2_, and 6.4 mL ultrapure water and digested for 1 h at 90°C using a Metrohm 705 UV Digester. The samples were then analyzed using AAS. A Perkin Elmer AAnalyst 800 instrument was used, either in flame or in graphite furnace mode, depending on the Ni concentrations. Calibration standards of 0, 1, 6, and 20 mg L^-1^ were used for the flame analysis. Samples spiked with known amounts of Ni ions revealed acceptable recoveries (80–110%) for all solutions and methods.

The calibration curves in cell medium and ALF were linear to approx. 6 mg L^-1^, with a deviation of approx. 10% from a linear extrapolation at 20 mg L^-1^. Based on the method by Vogelgesang and co-workers [24], the limit of detection (LOD) in cell medium was estimated to 0.11 mg L^-1^, the limit of identification (LOI) to 0.22 mg L^-1^ and the limit of quantification (LOQ) to 0.31 mg L^-1^. In ALF, the corresponding limits were 0.21, 0.42, and 0.69 mg L^-1^, respectively. For the graphite furnace measurements, calibration standards of 10, 30, 60, 100 and 200 μg L^-1^ were used. The calibration curve was linear up to a concentration of 100 μg L^-1^, and the deviation from the linear curve was 10% at 200 μg L^-1^. In cell medium, the LOD was estimated to 16 μg L^-1^, the LOI to 32 μg L^-1^, and the LOQ to 48 μg L^-1^. The corresponding limits in ALF were 16, 32 and 41 μg L^-1^, respectively. Blank solutions (without any particles) were analysed for all experiments. If the blank values exceeded the LOD, they were subtracted from the measured samples.

### Ni release into solution

Particle dispersions (10 μg mL^-1^) were prepared in cell medium and ALF. The particles were weighed directly into the vessels before sonication and the exact loading of particles for each experiment was hence known. The suspensions were incubated at bilinear shaking conditions (12°, 25 cycles/min, Stuart S180) for 4 and 24 h. The temperature was kept at 37°C during the incubation. To separate the particle fraction from the supernatant, the suspensions of the micron-sized particles were centrifuged for 10 min at 700 g. The nano-sized particles were treated with an ultracentrifugation method for 1 h (52900 g, Beckman Optima L-90K, SW-28 rotor). According to Tsao and co-workers [25], this procedure should remove all nano-sized particles from the suspension, considering the substantial agglomeration of particles in cell medium (Table 1). Triplicate samples were prepared.

**Table 1:** Particle size distributions and surface area measurements.

	Volume weighed (*μ*m)			Number weighed (*μ*m)			BET (m^2^ g^-1^)
**Particle**	d_0.1_	d_0.5_	d_0.9_	d_0.1_	d_0.5_	d_0.9_	
**Ni-n**	2.8 ± 1.4	3.4 ± 2.1	4.0 ± 2.8	0.11 ± 0.05	0.14 ± 0.09	3.0 ± 2.1	6.41
**NiO-n**	0.70 ± 0.01	0.82 ± 0.03	2.2 ± 1.7	0.17 ± 0.07	0.74 ± 0.02	0.88 ± 0.01	102^b^
**Ni-m1**	1.4 ± 0.46	2.8 ± 0.34	7.2 ± 0.12	0.90 ± 0.83	1.3 ± 0.51	2.4 ± 0.63	1.05^a^
**Ni-m2**	1.0 ± 0.98	1.8 ± 0.26	4.0 ± 0.74	0.72 ± 0.61	1.1 ± 0.75	1.8 ± 0.67	2.15^a^

Volume and number weighed particle sizes of Ni metal (Ni-n, Ni-m1 and Ni-m2) and Ni oxide (NiO-n) particles, after 1 h of incubation in cell medium (DMEM^+^), as well as particle specific surface areas (BET) at dry conditions. d_0.1_ = particle diameter at which 10% of particles are smaller; d_0.5_ = particle diameter at which 50% of particles are smaller (median); d_0.9_ = particle diameter at which 90% of particles are smaller.

^a)^ [18]

^b)^ [32]

### Oxidative reactivity

The ability of Ni and NiO particles to generate acellular (intrinsic) reactive oxygen species (ROS) was measured with the 2’7-dichlorodihydrofluorescin diacetate (DCFH-DA) assay, based on the description by Rushton and co-workers [26]. DCFH-DA is a non-fluorescent compound that is freely taken up by cells. It is hydrolyzed by intracellular esterases that remove the DA group, after which DCFH can be oxidized forming the fluorescent dichlorofluorescein (DCF). This oxidation may be induced by a transfer of electrons from a number of oxidative species, such as RO_2_^•^, RO^•^, OH^•^, HOCl and ONOO^-^ [27,28]. Whether or not H_2_O_2_ can oxidize DCFH appears to be controversial [27–30]. In the acellular assay, where cellular peroxidases are absent, this oxidation is commonly catalyzed by the addition of horseradish peroxidase (HRP). Particle suspensions were added on black clear bottom 96 well plates (25 μL/well), where DCFH with (+) or without (-) HRP was added (75 μL/well). Samples of all particle types containing PBS (75 μL/well), instead of DCFH, were included in each experiment for detecting any background fluorescence by the particles. The final Ni concentration was 20 μg mL^-1^. The plates were incubated at dark conditions (37°C, 1 h). Fluorescence was measured using 485 nm excitation and 530 nm emission wavelengths (Molecular Devices SpectraMax^®^ Gemini EM Microplate Reader). Each experiment was performed three times in triplicate wells.

Detection of intracellular ROS levels was performed by using the cellular DCFH-DA assay. A549 cells were seeded in black clear bottom 96 well plates and after 24 h exposed to Ni-n, NiO-n, Ni-m1 and Ni-m2 particles at a total Ni concentration of 20 μg cm^-2^. Nano-sized CuO (20 μg cm^-2^) and hydrogen peroxide (H_2_O_2_, 200 μM) were used as positive controls. After 2 h cells were loaded with 40 μM DCFH-DA in HBSS (Hank’s buffered salt solution) for 30 min at 37°C. Subsequently, cells were washed with HBSS and fluorescence was recorded every 5 min over 2 h (excitation 485 nm, emission 535 nm) using a plate reader (Victor^3^ V multilabel plate reader, Perkin Elmer). ROS increase was calculated as mean slope per min and normalized to the unexposed control. Results are presented as mean ± standard deviation of 3 independent experiments.

### Cell culture

Cells from a human type II alveolar epithelial cell line (A549, obtained from the American Type Culture Collection, ATCC, Manassas, USA) were cultured in DMEM (Dulbeccos´s Minimal Essential Medium, Cat. No. 41965–039, Gibco^®^ Invitrogen) cell culture medium supplemented with 10% Fetal Bovine Serum (European grade, Biological Industries), 1 mM Sodium Puryvate (Gibco^®^ Life Technologies), 100 units mL^-1^ Penicillin and 100 μg mL^-1^ Streptomycin (Pen Strep, Gibco^®^ Life Technologies). The supplemented medium is denoted as DMEM^+^. The cells were cultured in cell culture flasks in a humidified (RH > 99%) CO_2_-atmosphere (5%) at 37°C. The cells were seeded 24 h prior to each assay at concentrations of 0.08·10^6^, 0.04·10^6^ and 0.02·10^6^ cells cm^-2^ for 4, 24 and 48 h exposure times, respectively, in order for the (control) cells to reach confluence in the end of each exposure. CuO nanoparticles (20–40 nm diameter, Sigma-Aldrich), dispersed in DMEM^+^ at concentrations of 20 or 40 μg cm^-2^, were used as positive controls in all cellular assays.

### Cell viability

Cell viability was analyzed using the alamar blue assay. The assay indicates the cellular metabolic activity, which depends on the cell viability and on the number of cells (proliferation) in the culture. A549 cells were exposed to particle suspensions (in DMEM^+^), corresponding to total Ni concentrations of 0.1, 1, 5, 10, 20 and 40 μg cm^-2^, for 24 and 48 h in transparent 48 well plates. These concentrations equal to 0.1, 1, 5, 10, 20 and 40 μg mL^-1^. After exposure, the suspensions were removed and the cells were incubated in 200 μL of 10% alamarBlue^®^ (Invitrogen, Life Technologies) for 3 h. Fluorescence was measured using 560 nm excitation and 590 nm emission wavelengths (Molecular Devices SpectraMax^®^ Gemini EM Microplate Reader). Each experiment was performed three times in duplicate wells. Possible interferences between the particles and alamar blue were examined with a similar assay at cell-free conditions. Cell viability studies were also performed with the released fraction of Ni (S1 File, S2 Fig). In addition, cell viability was analyzed in terms of the cell membrane integrity with the trypan blue exclusion assay, as described by Midander and co-workers [17]. For this assay, the cells were exposed for 4 h to each particle type at a total Ni concentration of 20 μg cm^-2^.

### Colony forming efficiency

The clonogenic potential following exposure to Ni-n, NiO-n, Ni-m1 and Ni-m2 was studied by colony forming efficiency (CFE) assay. A549 cells were seeded at a density of 75 cells/mL in 2 mL culture medium in 6 well plates. After 24 h, particle suspensions were added directly to the cell cultures in order to obtain total Ni concentrations ranging from 0.1 to 40 μg cm^-2^. Untreated cells were used as a negative control and 40 μg cm^-2^ nano-sized CuO particles were used as a positive control. After 4 and 24 h exposures the cells were washed twice with PBS and fresh culture medium was added. After 3 days the medium was changed into fresh culture medium and after a total of 7 days cells were fixed with 3.7% (v/v) formaldehyde solution (Sigma-Aldrich) in PBS for 30 min and stained with 10% (v/v) Giemsa solution (Sigma-Aldrich) in deionized water for 30 min. Colonies were scored manually under a stereomicroscope. The results were normalized to the negative control and expressed as

$$\frac{{\left(average\;No.\;colonies\right)}_{\text{exp}osed}}{{\left(average\;No.\;colonies\right)}_{control}}x100$$

The corresponding Standard Error of the Mean (SEM) was calculated for 3 independent experiments. In each experiment were included 2 replicates for each treatment.

### DNA damage

The alkaline single cell comet assay was used for investigating the levels of DNA damage in A549 cells induced by Ni and NiO particles. In order to avoid artifacts caused by excess cytotoxicity, a suitable Ni concentration for the assay (20 μg cm^-2^) was chosen based on the cell viability tests. Cells were exposed to particle suspensions for 4 and 24 h in 24 well plates. The comet assay was performed as described previously [31]. At least 100 cells were analyzed from each sample. The extent of DNA damage was measured as the % DNA in tail, which represents the fraction of the total DNA that is contained in the comet tail.

### Cellular uptake and quantification of cell-associated Ni-fraction

In order to investigate particle uptake and intracellular localization as well as particle dissolution in lysosomes, cells were analyzed using TEM-imaging. A549 cells were seeded in 6 well plates and 24 h later exposed to Ni-n, NiO-n, Ni-m1 and Ni-m2 particles at a total Ni concentration of 20 μg cm^-2^ for 4 h. After exposure, the cells were thoroughly washed and either harvested or cultured for further 24 h in fresh cell culture medium in order to evaluate the particle dissolution in the cells. Cell samples were fixed in 0.1 M glutaraldehyde solution and the TEM grids were prepared as previously described [31].

The total amount of Ni that was taken up by the cells or bound to the cell membrane during exposure was also analyzed quantitatively using AAS. A549 cells were exposed to particle suspensions, corresponding to a total Ni concentration of 20 μg cm^-2^ (total Ni mass of 40 μg) for 4 h in 24 well plates. After the exposure, the supernatant was discarded and the cells were washed with 3 x 1 mL PBS. The washed cells were harvested with 20 μL Trypsin, and suspended in 200 μL DMEM^+^. Cell suspensions were centrifuged (210 g, 4 min, 20°C), the supernatant was removed, and the cell pellet was re-suspended in PBS (200 μL). The final cell concentration was counted using a Bürker chamber, after which the remaining cell suspensions were acidified with 0.5 mL 65% HNO_3_. The cell-associated Ni-fractions were quantified using AAS (as described under “Ni concentration determination”). The percentage of Ni that was either taken up by the cells or bound to the cell membrane was calculated based on the total measured mass of the cell-associated Ni and the total mass of Ni (40 μg) that was initially applied onto the cells.

### Statistical analysis

Statistical analyses were performed in R (version 3.1.1, R Core Team 2014). Data was analyzed with one-way analysis of variance (ANOVA). In cases where the presumptions of ANOVA were not met, the data was analyzed with the non-parametric Kruskal-Wallis analysis of variance. Tukey HSD test was used for post-hoc testing. The level of statistical significance was set to 0.05. All results are expressed as the mean value ± the standard deviation (SD). All measurements were performed in three individual replicates (n = 3).

## Results

### Particle morphology and size

**Fig 1:**
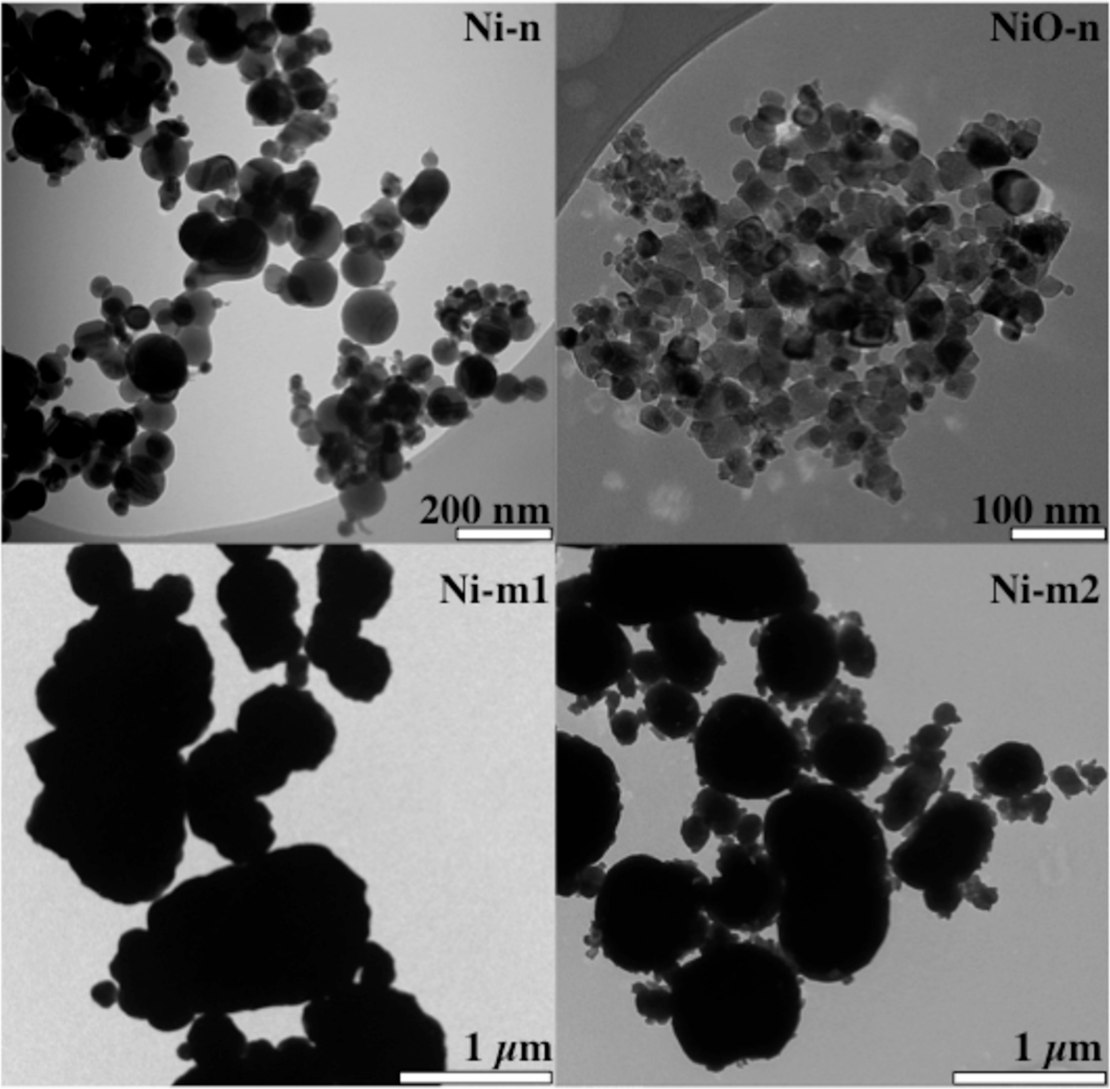
Primary size and morphology of the particles. Nano- and micron-sized nickel metal (Ni-n, Ni-m1, Ni-m2) and nickel oxide particles (NiO-n) recorded with Transmission Electron Microscopy (TEM).

Transmission electron microscopy (TEM) images of the different Ni and NiO particles are shown in Fig 1. The median particle sizes in cell medium, and the specific surface areas (BET) at dry conditions are presented in Table 1. The results show clearly that each particle type agglomerates to a different extent in cell medium. This makes the size differences between the micron- and the nano-sized particles smaller when compared to their corresponding primary particle sizes (Fig 1 and Table 1). All particles formed polydisperse agglomerates in sizes between several hundred nanometers and several microns.

### Ni release into solution

**Fig 2:**
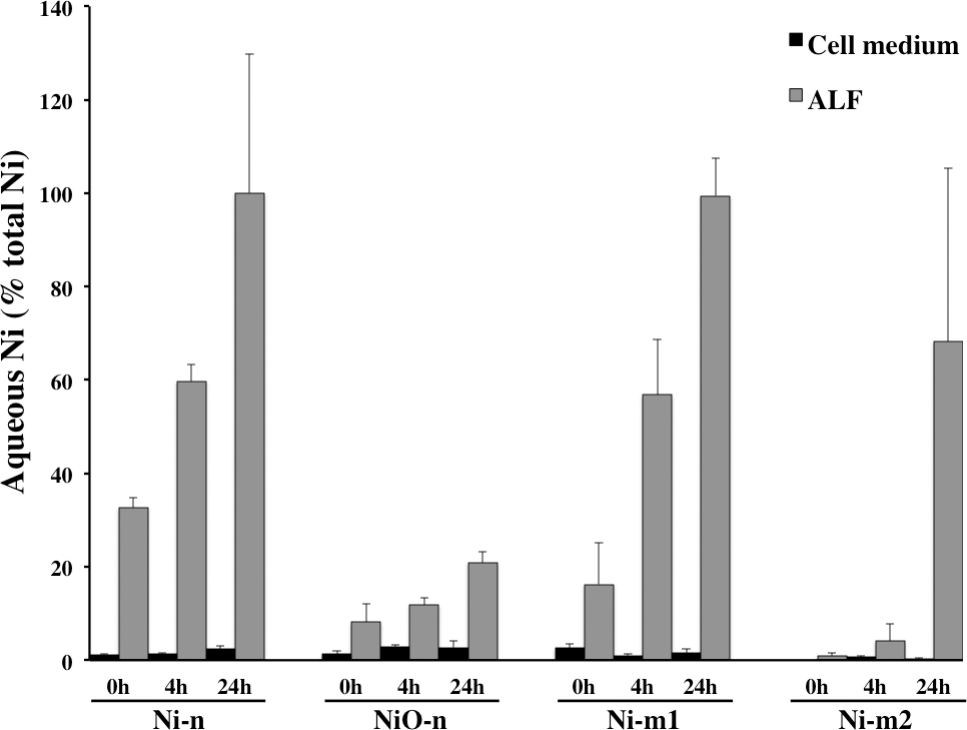
Ni release into solution. Released amount of Ni in solution (aqueous Ni) compared with the total amount of Ni in the particles (Ni-n, Ni-m1, Ni-m2 and NiO-n). Release was analyzed after 0, 4 and 24 h incubation of the particle suspensions in cell medium or ALF (analyzed with AAS). Each bar represents the mean value of three independent experiments (n = 3), and the error bars the standard deviation of the mean value (±SD). Results for “0 h” correspond to measurements made directly after sonication of the particle dispersions, and therefore represent the starting point of the cell exposures.

The amount of Ni released into solution from Ni and NiO particles was significantly higher and more rapid in ALF than in cell medium (Fig 2). In ALF, Ni was released most rapidly from Ni-n and Ni-m1. Ni release from these particles corresponded to complete (100%) particle dissolution after 24 h. In comparison, Ni release from NiO-n and Ni-m2 after 24 h in ALF was 21 and 68%, respectively. In cell medium the highest Ni release was observed for Ni-n, NiO-n and Ni-m1 (Fig 2). For these particles, the proportion of released Ni was approx. 1–3% after all exposure times (0, 4 and 24 h). The sometimes high standard deviations seen for the results in Fig 2 could to some extent depend on the variations in size distributions between samples (Table 1).

### Oxidative reactivity

**Fig 3:**
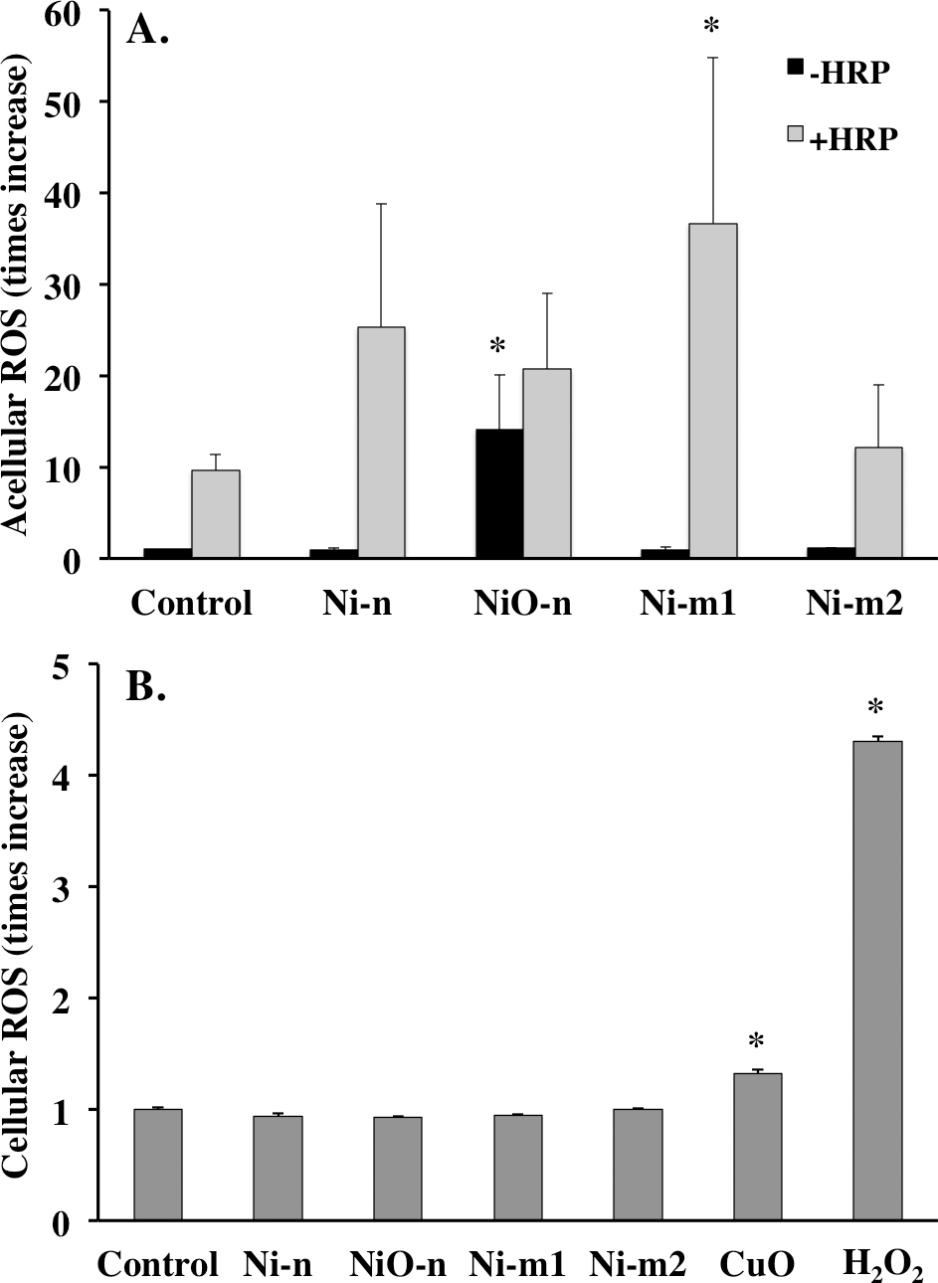
Oxidative reactivity. (A) Acellular ROS production of Ni metal (Ni-n, Ni-m1 and Ni-m2) and Ni oxide (NiO-n) particles (20 μg mL^-1^ of total Ni) studied with the acellular DCFH-DA assay in the presence (+HRP) and absence (-HRP) of a catalyst (Horse Radish Peroxidase). The oxidative reactivity is presented as the change in fluorescence intensity when compared with control (PBS with DCFH). (B) Intracellular ROS production in A549 cells exposed to Ni metal (Ni-n, Ni-m1 and Ni-m2) and Ni oxide (NiO-n) particles (20 μg mL^-1^ of total Ni) using the cellular DCFH-DA assay. Nano-sized CuO and H_2_O_2_ were used as positive controls. The ROS increase was calculated as mean slope per min and normalized to the unexposed control. The asterisk (*) assigns statistically significant (p<0.05) values compared with the corresponding control (PBS with DCFH ±HRP or unexposed cells).

The intrinsic ability of Ni and NiO particles to generate ROS was studied with the 2’7-dichlorodihydrofluorescin diacetate (DCFH-DA) assay. In the presence of a catalyst (+HRP), Ni-m1 was the most reactive particle. It induced an almost 37-fold increase in ROS generation, compared to the basal level (PBS treated with DCFH). This increase was statistically significant when compared with the corresponding control (PBS treated with DCFH +HRP) (Fig 3). Additionally, Ni-n and NiO-n induced slight increases in ROS generation in the presence of HRP (Fig 3). Interestingly, NiO-n induced a significant (14-fold) increase in ROS generation in the absence of the catalyst (-HRP). In this case NiO-n was clearly the most reactive particle (Fig 3). The remaining particles did not induce notable increases in ROS generation in the absence of HRP. None of the particles affected the levels of background fluorescence, when investigated in PBS.

Unlike acellular ROS production, cellular ROS was not increased in A549 cells by exposure to any of the tested particles (Fig 3). In contrast, a clear increase was observed following exposure to the positive particle control (CuO) and H_2_O_2_. Thus, at the time point and concentration tested, the cells were protected from Ni particle induced oxidative stress.

### Cell viability

**Fig 4:**
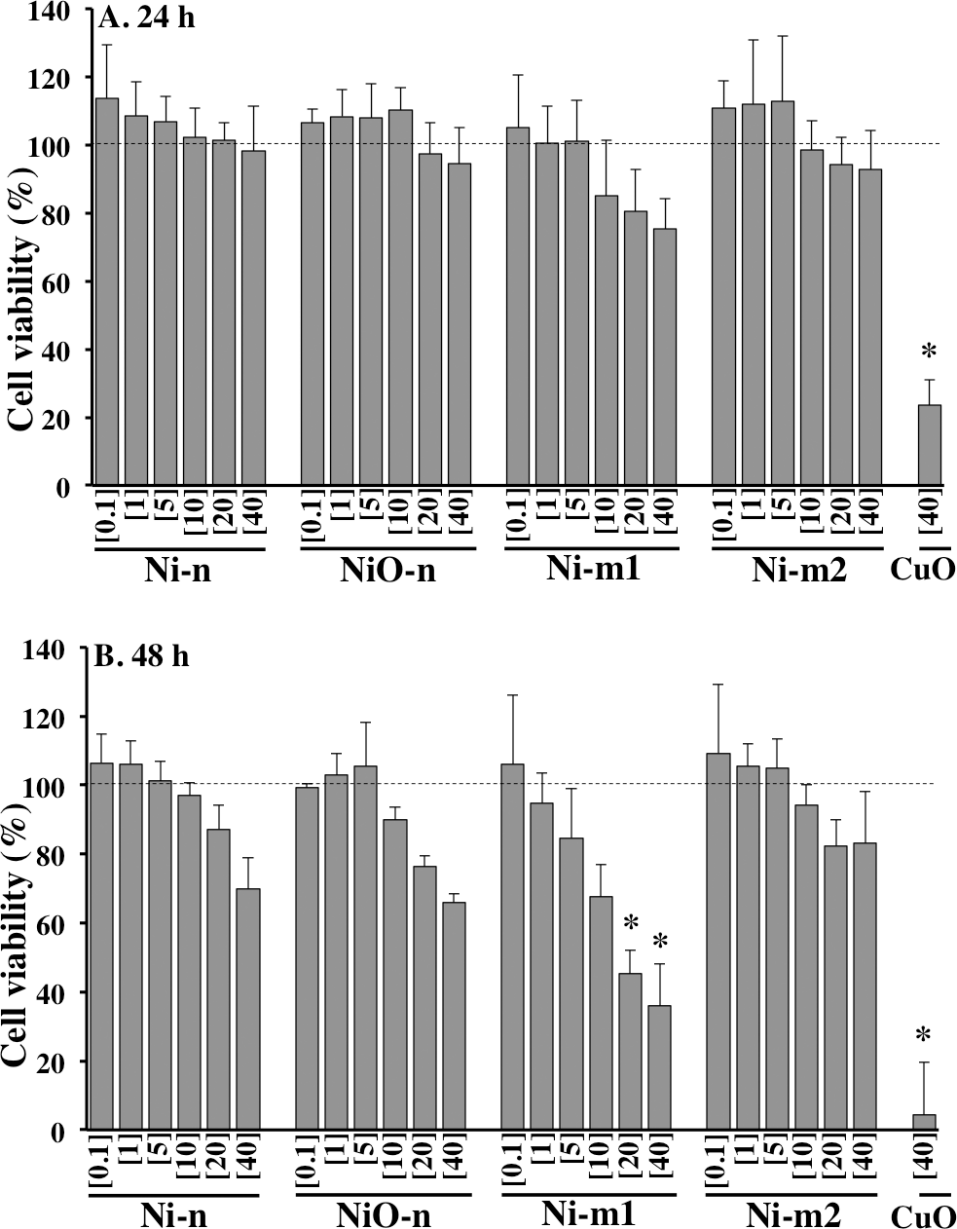
Cell viability of A549 cells. (A) 24 h and (B) 48 h exposure to Ni metal (Ni-n, Ni-m1 and Ni-m2) and Ni oxide (NiO-n) particle suspensions at total Ni concentrations of 0.1, 1, 5, 10, 20 and 40 μg cm^-2^. CuO-nanoparticle suspensions (40 μg cm^-2^) were used as positive controls. Each bar represents the mean value of three independent experiments (n = 3), and each error bar the standard deviation of the mean (±SD). The asterisk (*) assigns statistically significant (p<0.05) values.

The influence of Ni and NiO particles on A549 cell viability was measured in terms of cellular metabolic activity (Fig 4). The most distinct effect was the dose dependent decrease in cell viability by Ni-m1 in the entire concentration range (0.1–40 μg cm^-2^ of total Ni). After 48 h the cell viability was reduced to 45 and 36% at the two highest Ni concentrations (20 and 40 μg cm^-2^), respectively. These reductions were statistically significant (p<0.05). Cell viability was also reduced after 48 h by Ni-n, NiO-n and Ni-m2 at the highest Ni concentrations, although, these effects were not statistically significant. Interestingly, an enhanced cellular metabolic activity was observed at the lowest Ni concentrations of each particle suspension. This is presumably a consequence of an increased cell number, and thus a sign of a proliferative effect. Although the effect was observable, it was not statistically significant. The particle suspensions did not cause interference with alamar blue.

In order to investigate whether the observed effects on cell viability were related to extracellular released Ni in cell medium, additional cell viability tests were performed using the released Ni fractions, from which the particles had been separated. The released Ni fractions did not induce any detectable effects on cell viability (S2 Fig). Furthermore, there were no major effects on cell membrane integrity after 4 h exposure to the particle suspensions. However, a slight, but non-significant, reduction in cell viability (90.4%) was observed for NiO-n (S3 Fig).

### Colony forming efficiency

**Fig 5:**
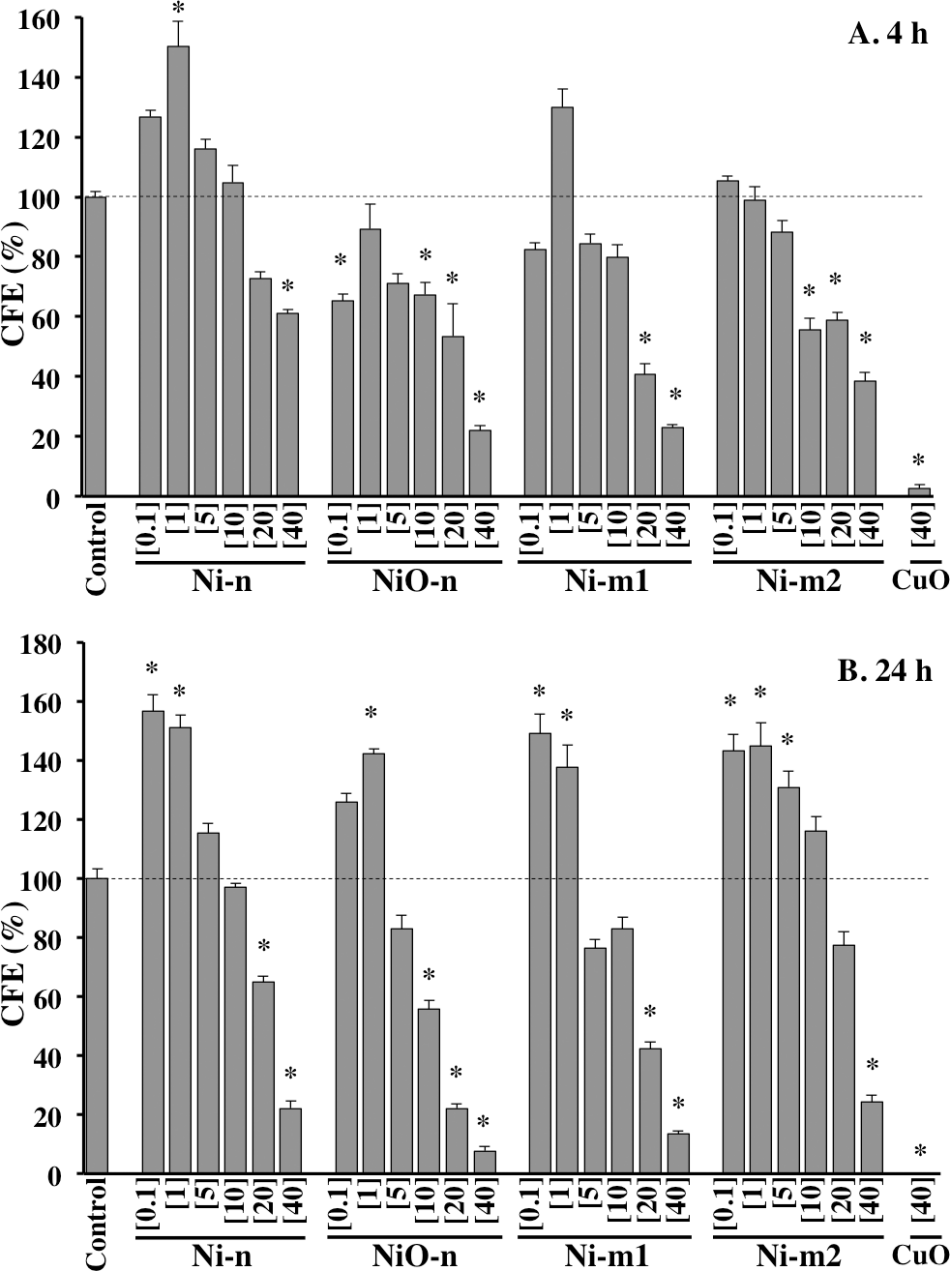
**Colony forming efficiency** (CFE) after (A) 4 h and (B) 24 h exposure (and 7 days post-incubation) to Ni metal (Ni-n, Ni-m1 and Ni-m2) and Ni oxide (NiO-n) particle suspensions at total Ni concentrations of 0.1, 1, 5, 10, 20 and 40 μg cm^-2^. CuO-nanoparticle suspensions (40 μg cm^-2^) were used as positive controls. Each bar represents the mean value of three independent experiments (n = 3), and each error bar the standard error of the mean (±SEM). The asterisk (*) assigns statistically significant (p<0.05) values.

Due to the sign of a weak proliferative effect of low Ni concentrations in the cell viability assay, this response was studied further with the colony forming efficiency (CFE) assay. While each of the particles was cytotoxic at the highest concentrations after 4 h of exposure, Ni-n induced a significant increase in CFE, indicating increased cell proliferation, at the concentration of 1 μg cm^-2^ (Fig 5). Furthermore, after 24 h of exposure cell proliferation was increased significantly by each of the particles at least at one of the lowest concentrations (between 0.1 and 5 μg cm^-2^ of total Ni).

### DNA damage

**Fig 6:**
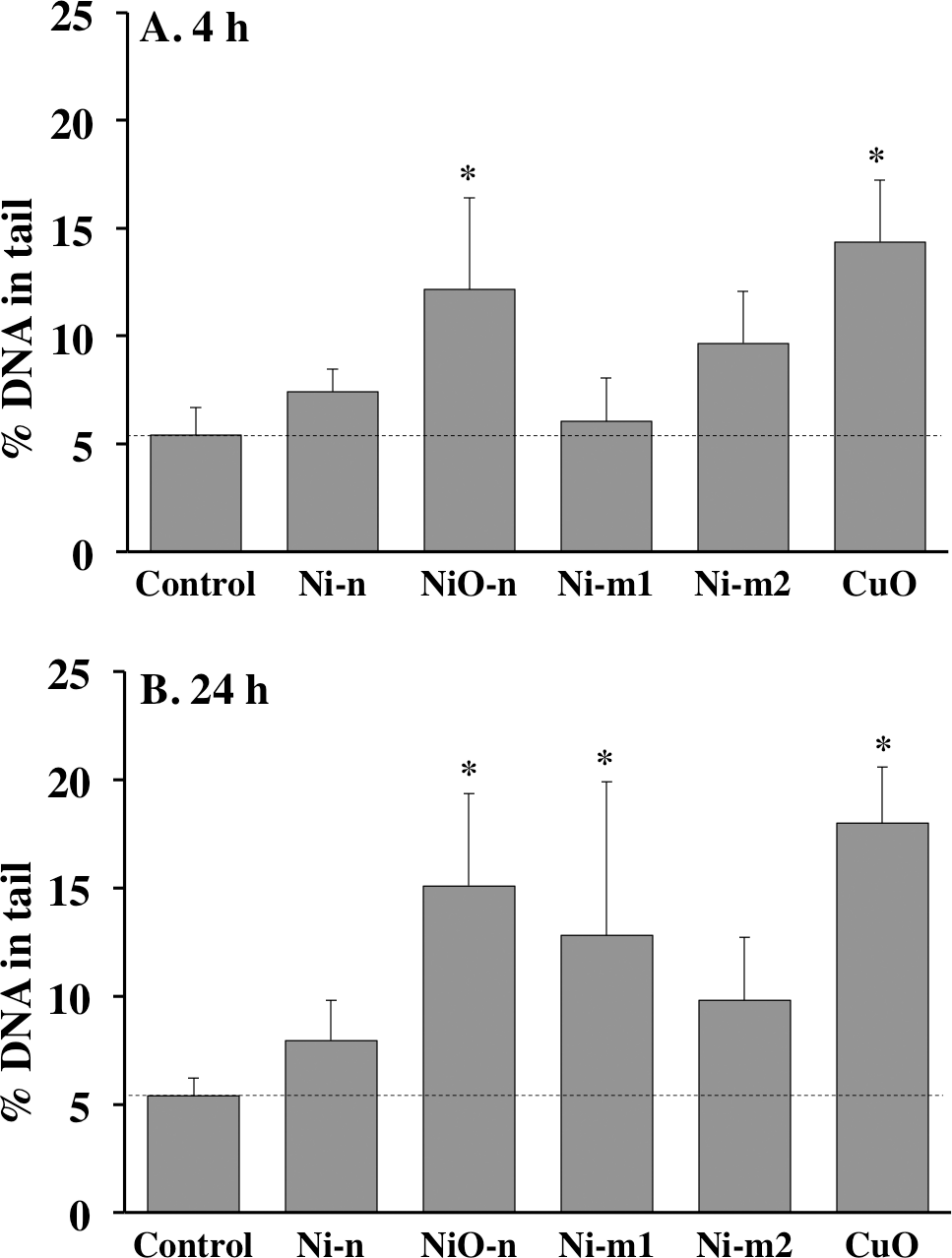
DNA damage in A549 cells. DNA damage analyzed with the comet assay after (A) 4 h and (B) 24 h of exposure to Ni metal (Ni-n, Ni-m1 and Ni-m2) and Ni oxide (NiO-n) particle suspensions (20 μg cm^-2^ of total Ni). Cells exposed to CuO-nanoparticle suspensions (20 μg cm^-2^) were used as a positive control. The asterisk (*) is assigned for statistically significant (p<0.05) values. Each bar represents the mean value of three independent experiments (n = 3), and the error bars the standard deviation of the mean value.

The level of DNA damage in A549 cells induced by Ni and NiO particles was analyzed with the alkaline single cell comet assay. NiO-n was the most potent particle inducing DNA damage of 12.4 and 15.1% (DNA in tail) after 4 and 24 h exposures, respectively (Fig 6A and 6B). These levels were 2–3 times higher, and statistically significant, when compared to the control cells (5.4% DNA in tail). Additionally, Ni-m1 induced a significant increase in DNA damage (12.8% DNA in tail) after 24 h, but not after 4 h exposure. Ni-n and Ni-m2 induced slightly increased, however, non-significant DNA damage, in both exposure times, but especially after 24 h.

### Cellular uptake and quantification of cell-associated Ni-fraction

**Fig 7:**
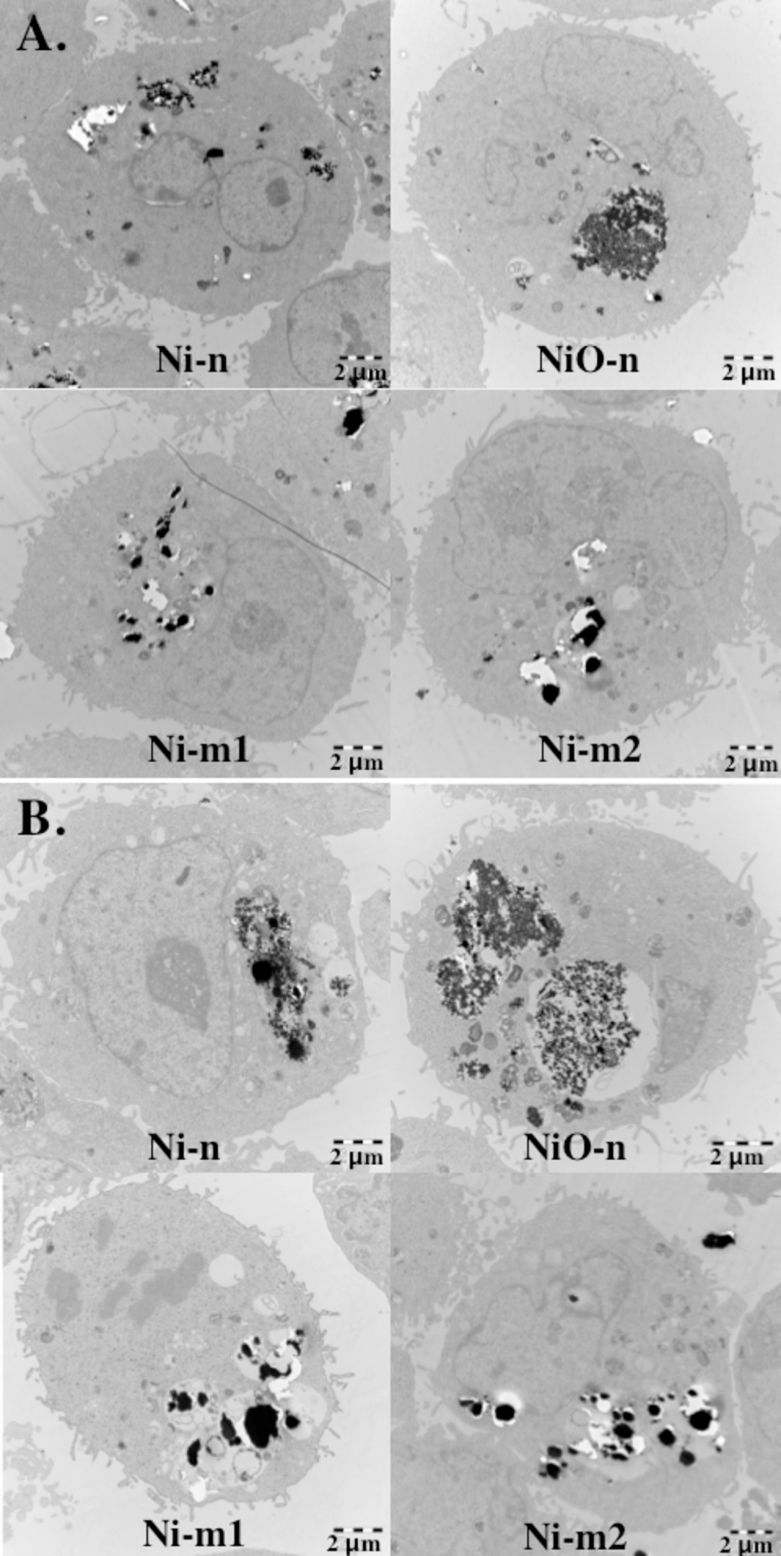
Particle uptake and intracellular localization. A549 cells exposed to nano- and micron-sized nickel metal (Ni-n, Ni-m1, Ni-m2) and nickel oxide particles (NiO-n) recorded with Transmission Electron Microscopy (TEM) (A) directly after or (B) 24 h after a 4 h exposure (20 μg cm^-2^ of total Ni).

When cellular uptake of the particles was investigated with TEM, it was visually confirmed that the cells internalize each of the four particle types (Fig 7). As this method is merely qualitative, the amount of uptake between the particles could not be differentiated. The particles were mostly observed to be localized in endosome-like structures. The amount of intracellular particles did not appear to diminish after a post-exposure time of 24 h. This suggests that the intracellular Ni release from these particles is considerably lower than the almost complete dissolution observed in ALF following 24 h (Fig 2).

**Fig 8:**
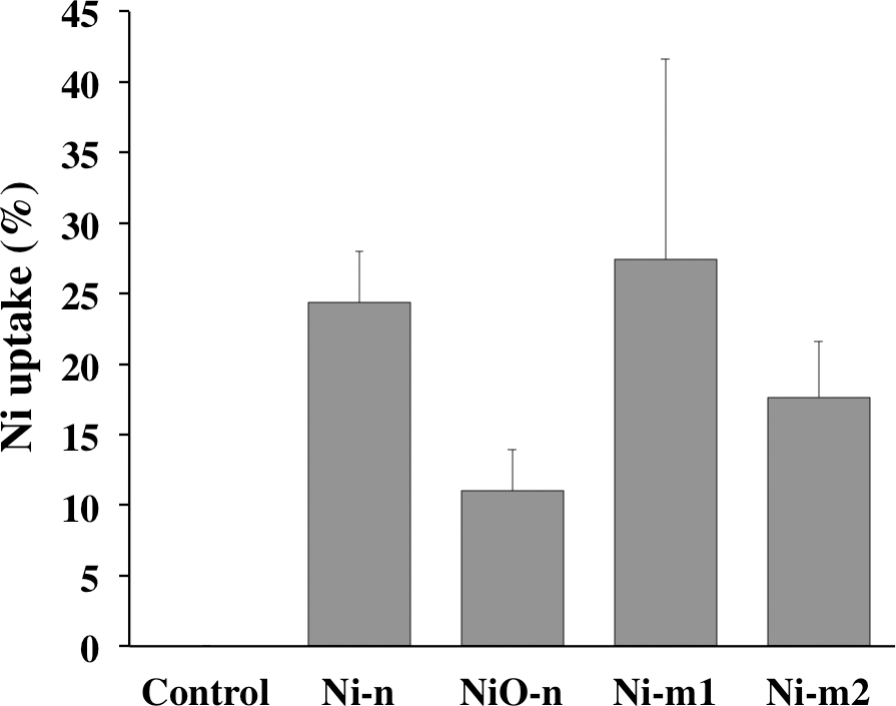
A549 cell-associated Ni-fraction. The amount of Ni that was taken up by the cells or bound to the cell membrane was analyzed with AAS after 4 h of exposure to Ni metal (Ni-n, Ni-m1 and Ni-m2) and Ni oxide (NiO-n) particle suspensions (20 μg cm^-2^ of total Ni). The cell-associated Ni-fraction is presented as the percentage of the total amount of added Ni in the exposure suspensions. Each bar represents the mean value of three independent experiments (n = 3), and the error bars the standard deviation of the mean value.

The cell-associated Ni-fraction in A549 cells was defined as the total amount of Ni, both as particles and as released Ni species, that was taken up by the cells, or was strongly bound to the cell membrane at the time point when the exposure was terminated (4 h). Exposure to each of the Ni and NiO particle suspensions caused considerably increased levels in the cell-associated Ni, when compared to the background Ni levels in the control cells (Fig 8 and S4 Fig). The fraction of cell-associated Ni was higher than 10% of the total mass of added Ni for each of the studied particles (Fig 8).

## Discussion

The aim of this study was to investigate and compare the characteristics of nickel metal (Ni) and nickel oxide (NiO) particles with a focus on Ni release and ROS generation, cellular uptake, cytotoxicity and genotoxicity. A compilation of the results is presented in Table 2.

**Table 2:** Compilation of the responses of Ni metal (Ni-n, Ni-m1 and Ni-m2) and Ni oxide (NiO-n) particles to different assays in this study.

**Particle**	**Ni release, cell medium**	**Ni release, ALF**	**Oxidative reactivity**	**Cellular dose**	**Cell viability**	**CFE**	**DNA damage**
**Ni-n**	1	4	2	4	3	4	1
**NiO-n**	1	3	4	2	3	2	4
**Ni-m1**	1	4	4	4	4	3	3
**Ni-m2**	0	3	0	3	3	3	2

Results of each assay have been normalized to the corresponding findings for the particle with the highest response (4). A zero (0) represents a response that did not differ from the control. Here, the cellular dose is defined to include both particles and released Ni species firmly attached to the cell membrane, as well as internalized into the cells.

The results of this study show that Ni release into solution by each of the Ni and NiO particles was considerably higher in ALF than in cell medium. For example, two particles (Ni-n and Ni-m1) underwent a rapid and complete (100 wt%) dissolution within 24 h in ALF (Fig 2). This is likely related to the combined effect of a relatively low pH (4.5) and the presence of Ni-complexing agents in ALF. Similar conditions have been shown to enhance Ni release from stainless steel particles [33]. Our results for Ni-m1 and Ni-m2 are also in line with previous observations for these particles [18]. Furthermore, adsorption of proteins on metal surfaces and protein-metal complexation in solution enhances the release of metals from stainless steel in a similar way [34]. According to Hedberg and co-workers, this enhancement is in particular related to ligand-induced metal release mechanisms [33,35]. However, depending on the metal and the adsorbed ligands, the release may also be hindered [34]. The considerably lower Ni release in cell culture medium (ca. 1–3 wt%) is in line with a previous study on NiO-n [5]. Although Pietruska and co-workers, conversely, reported 40% Ni release in cell culture medium for NiO-n, they showed <0.5% release for Ni-n and minor release for micron-sized Ni [19].

In order to link these acellular assays to the cellular *in vitro* conditions, the particle uptake and intracellular dissolution was studied using TEM-imaging. Compared to the quantitative chemical analysis of Ni release, this method is qualitative. It can be used to validate particle uptake and merely give an indication of possible intracellular dissolution. Each of the particles was clearly taken up by the cells within 4 h of exposure. Thereafter, the particles remained in the cells and appeared to be largely non-dissolved after a 24 h post-incubation, suggesting that the Ni release in ALF did not reflect the intracellular Ni release *in vitro* in this study. This is an interesting observation, taking into account the importance of Ni uptake and the role of intracellular Ni release for the toxicity of Ni-containing particles [7,36]. Our results suggest that intracellular Ni release from the four studied particles is relatively slow, which may result in a persistent intracellular exposure to low levels of Ni.

Cell viability was only affected by the particle suspensions (containing both particles and the released Ni fraction) and not by the released Ni in cell medium (Fig 5, S1 Fig). Although cytotoxic effects by extracellular released Ni have been reported previously [20], this was not observed in our study (S1 Fig). Reasons why the released Ni fractions did not affect cell viability are most likely related to the relatively low Ni release in cell medium (Fig 2) and possibly to a weak cellular uptake of the released Ni species. For example, chemical speciation modeling suggests that released Ni in cell medium forms stable complexes with different ligands such as amino acids (S2 File, S2 Table, S5 Fig). Similar effects on cell viability have also been reported by Cho and co-workers [37]. Comparably to our study, they found that nano-sized NiO particles, but not the released Ni fraction, affected A549 cell viability (24 h exposure) [37]. Interestingly, they found similar effects also *in vivo*; the instillation of NiO particles into rat lungs caused an acute (24 h) inflammation that was observed to advance over the course of four weeks, while the released Ni fraction did not cause any inflammatory responses [37]. Based on our results as well as the previous studies, it is concluded unlikely that extracellular released Ni would contribute notably to the observed toxicity of Ni and NiO particles. Therefore, these results seem to support a theory of a Trojan-horse type mechanism and the “Ni ion bioavailability” model for Ni and NiO particles [7].

As genotoxicity is regarded as an important endpoint for carcinogenicity, we compared the potential of the Ni and NiO particles to induce DNA damage by using the comet assay. DNA damage after 4 h was most pronounced by exposure to NiO-n (Fig 6). Also the remaining particles induced slightly increased DNA damage, but mostly after 24 h. NiO-n was also reactive in terms of acellular ROS generation (Fig 3). In relation to the other particles, it was especially reactive in the absence of a catalyst (-HRP). This relative difference, however, changed when the catalyst was added (+HRP). In these conditions, Ni-m1 generated the highest levels of ROS, and also Ni-n was reactive. However, intracellular ROS in A549 cells was not increased by any of the particles at the dose and time point tested. These seemingly different responses between the acellular and cellular assays might be due to the adsorption of biomolecules on the particles in cell culture medium and inside the cells. For instance, some chelators have previously been shown to reduce the generation of hydroxyl radical (OH^•^) by Ni^2+^ [38]. However, other reports conclude that unlike for many other redox-reactive metals, ligand binding may in fact promote the oxidation of Ni (from Ni^2+^ to Ni^3+^) [39]. The observed difference between the acellular and the cellular assay could also be due to the antioxidative defense mechanisms of the cells and their ability to counteract the intrinsic oxidative reactivity of some of the particles. Additionally, the A549 cells may be particularly resistant to oxidative stress [5]. Our results for NiO-n are in line with previous studies, where these particles caused DNA damage in lung cells (A549 and BEAS-2B) [32] as well as in mouse embryonic stem cells (mES) [15]. In the latter study, NiO-n also induced acellular ROS production (-HRP) as well as an induction of an oxidative stress reporter [15,32]. In general, previous studies using the DCFH-DA assay to assess intracellular ROS in A549 show mixed outcomes; a study by Capasso and co-workers [5] reported negative results, while positive results were reported in two studies using higher doses compared to the ones tested in the present study [32,40]. Furthermore, other studies investigating ROS production from soluble Ni have found negative results when using A549 cells [41,42]. Additionally, when the toxicity of NiO-n is compared to CuO-n (positive control), an interesting observation can be made from a comparative cancer risk perspective; both particles induced DNA damage, but CuO-n was clearly more cytotoxic. The lower cytotoxicity of NiO-n may therefore imply a higher persistence of the induced DNA damage. This may further be an implication of the relatively low oxidative reactivity of Ni compared to more redox-active metals, such as Cu [43].

Differences in the observed responses between the particles become interesting when the three Ni metal particles (Ni-n, Ni-m1 and Ni-m2) are compared (Table 2). Surprisingly, the micron-sized Ni-m1 generated more acellular ROS and was more toxic than the nano-sized Ni-n. This is contrary to the common assumption that particle reactivity and toxicity increases along with a decreasing particle size and an increasing surface area [19,40]. According to the specific surface area (BET) measurements, also Ni-m2 has a larger surface area per mass than Ni-m1 at dry conditions, and is thus smaller than Ni-m1 (Table 1). It should be noted, however, that the studied particles agglomerate rapidly in solution (Table 1). This reduces the differences between the hydrodynamic particle sizes of the micron- and nano-sized particles, when compared to their primary particle sizes (Fig 1). Due to the relatively small differences in the cell-associated Ni-fraction of Ni-n, Ni-m1 and Ni-m2 (Fig 8), particle uptake cannot explain the observed differences in toxicity. This conclusion was confirmed by TEM-imaging (Fig 7). Our data therefore suggests that apart from the size, surface area and the chemical composition of these particles, there might be other factors affecting both their reactivity and toxicity. Although differences in particle uptake could not be established in our study, several characteristics in addition to particle size affect the uptake of Ni, as discussed in a review by Muñoz and Costa [36]. For example, the extent to which a particle can release Ni depends not only on the intrinsic bulk material properties and particle size, but also on the nature of the surface oxide, both in terms of composition and degree of crystallinity [18]. The studied particles have indeed several other characteristics that are distinctive for each particle type. The structure of the surface oxide is one of these traits; the surface oxide of Ni-m1 is composed of non-stoichiometric nickel oxide, *e*.*g*. Ni_2_O_3_ in the outer layer, whereas the oxide on Ni-m2 is thicker and primarily composed of NiO and of Ni(OH)_2_ and/or Ni_2_O_3_ [18]. Thus, it is believed, that the different surface oxides of Ni-m1 and Ni-m2 may contribute to the observed differences in Ni release and acellular ROS generation, as well as to the differences in toxicity. This hypothesis is supported by the fact that the conductance and valence band levels of Ni_2_O_3_ overlap with redox potentials of biological reactions [44]. This suggests that, due to the abundance of Ni_2_O_3_ on Ni-m1, the cellular redox state can be disturbed, which can possibly lead to oxidative stress. A similar effect is more unlikely for Ni-m2, which has a surface mostly composed of NiO that does not cause such overlap.

Another interesting aspect of Ni exposure is the enhanced cell proliferation at low Ni concentrations. This is clearly demonstrated in the results of this study (Figs 4 and 5). Although not statistically significant, an increased mitochondrial activity in the alamar blue assay, that might indicate an increased cell proliferation, was observed for each of the studied particles at the lowest Ni concentrations. A more distinct effect was observed with the colony forming efficiency assay (CFE). Each of the particles enhanced the proliferation of A549 cells significantly after 24 h of exposure at the low doses (0.1 or 1 μg cm^2^ of total Ni). As each of the particles induced significant cytotoxicity at the highest concentrations, these results suggest that Ni-containing particles may act via concentration-specific mechanisms (Fig 5). Due to the relevance of low Ni concentrations in real exposure environments, it should be underlined that the enhancement of proliferation may be a particularly important factor for Ni carcinogenicity in humans, and should thus be further assessed. A pathway leading to this effect was described in a recent study, where low Ni concentrations were shown to activate NADPH oxidase (nicotinamide adenine dinucleotide phosphate-oxidase) [16]. Additionally, ROS and a disrupted cellular redox-balance have been linked to proliferative effects [45].

## Conclusions

In this study we found a micron-sized Ni-m1 to be more reactive and toxic than nano-sized Ni-n and another micron-sized particle, Ni-m2. Thus, it is concluded that nano-sized Ni particles are not more toxic *per se* than micron-sized Ni. Instead, it is hypothesized that the reactivity and toxicity of Ni particles are strongly affected by the distinct differences in their surface oxide characteristics. However, the nature and the specific role of these characteristics remain to be further scrutinized. In addition to decreased cell viability in high Ni doses (≥10 μg cm^-2^) the colony forming efficiency assay indicated increased cell proliferation, compared to control, in low Ni doses (≤1 μg cm^-2^) by each of the studied particles. This highlights the need of future studies to take into account low Ni doses and the possibility for dose-specific mechanisms.

## Supporting Information

S1 Fig Measured concentrations of total Ni. Ni metal (Ni-n, Ni-m1, Ni-m2) and Ni oxide (NiO-n, NiO-m) particle dispersions with nominal nickel concentration of 20 μg cm^-2^. Each bar represents the mean value of three independent experiments.
(TIF)

S2 Fig Effect of released Ni fractions on cell viability. Cell viability of cultured A549 cells after exposure to released fractions of Ni (particles excluded) for initial Ni concentrations of 0.1, 1, 5, 10, 20 and 40 μg cm^-2^ after 24 (a) and 48 h (b) of exposure compared to the control (100%). Cell exposure to released fraction from CuO (40 μg/cm^-2^) was used as a positive control for the assay. Bars represent mean values of three independent experiments (n = 3). Error bars represent the standard deviation of the mean value (±SD).
(TIF)

S3 Fig Cell viability assessed via membrane integrity. Cultured A549 cells after 4 h exposure to particle dispersions of Ni metal (Ni-n, Ni-m1, Ni-m2) and Ni oxide (NiO-n) of a total Ni concentration of 20 μg cm^-2^. Cells exposed to CuO-nanoparticle dispersion (20 μg cm^-2^) were used as positive control. Each bar represents the mean value of three independent experiments.
(TIF)

S4 Fig Cellular dose of Ni. Cellular dose presented as the amount of Ni/cell. A549 cells after 4 h exposure to Ni metal (Ni-n, Ni-m1, Ni-m2) and Ni oxide (NiO-n) particle dispersions at total Ni concentration of 20 μg cm^-2^. Each bar represents the mean value of three independent experiments.
(TIF)

S5 Fig Ni speciation in solution. JESS solution speciation predictions of Ni in cell medium (DMEM; 10 μg mL^-1^). Abbreviations: Tyr = Tyrosine, His = Histidine, Gln = Glutamine, Thr = Threonine, Met = Methionine.
(TIFF)

S1 Table Chemical composition of Artificial Lysosomal Fluid (ALF). The concentration of each compound that was contained in ALF is listed in this table.
(DOCX)

S2 Table Chemical composition of DMEM in JESS modeling. The concentration of each compound in DMEM that was used in the JESS solution speciation predictions for Ni.
(DOCX)

S1 File Cell viability upon released nickel exposure. (DOCX)

S2 File Chemical equilibrium modeling. (DOCX)
