# Supplementary figures and images for: Nickel Release, ROS Generation and Toxicity of Ni and NiO Micro- and Nanoparticles
Source: PLoS One. 2016 Jul 19;11(7):e0159684. doi: 10.1371/journal.pone.0159684 (PMC4951072; doi:10.1371/journal.pone.0159684)

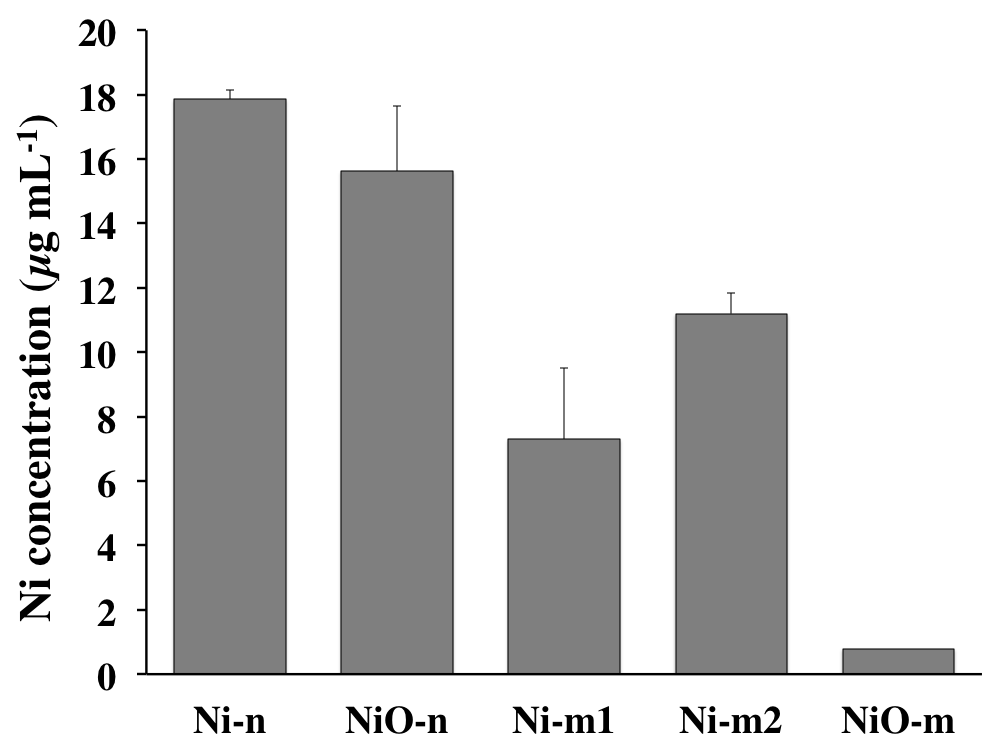

Supplement: S1 Fig — Ni metal (Ni-n, Ni-m1, Ni-m2) and Ni oxide (NiO-n, NiO-m) particle dispersions with nominal nickel concentration of 20 μg cm-2. Each bar represents the mean value of three independent experiments. (TIF) [file pone.0159684.s001.tif]

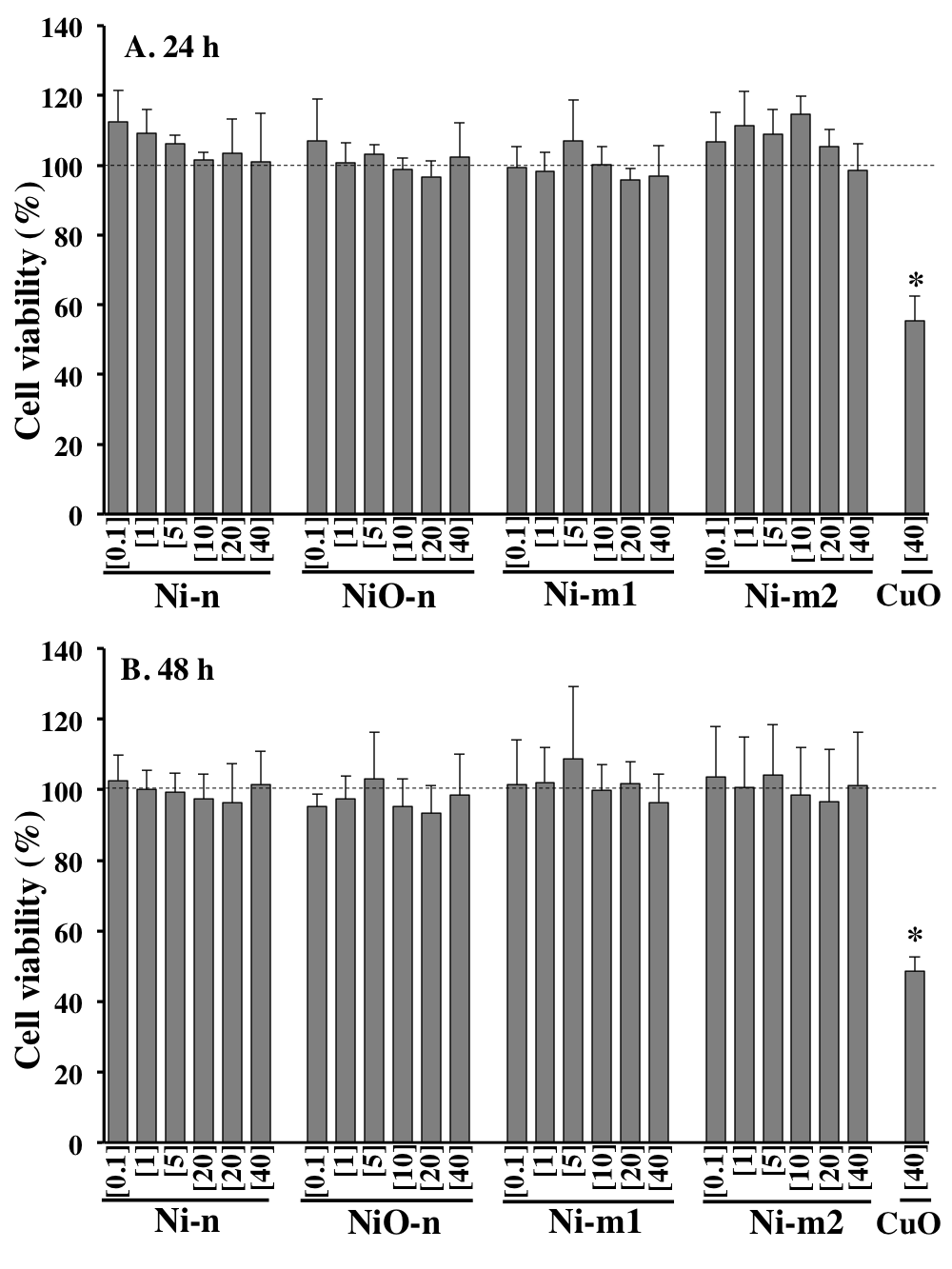

Supplement: S2 Fig — Cell viability of cultured A549 cells after exposure to released fractions of Ni (particles excluded) for initial Ni concentrations of 0.1, 1, 5, 10, 20 and 40 μg cm-2 after 24 (a) and 48 h (b) of exposure compared to the control (100%). Cell exposure to released fraction from CuO (40 μg/cm-2) was used as a positive control for the assay. Bars represent mean values of three independent experiments (n = 3). Error bars represent the standard deviation of the mean value (±SD). (TIF) [file pone.0159684.s002.tif]

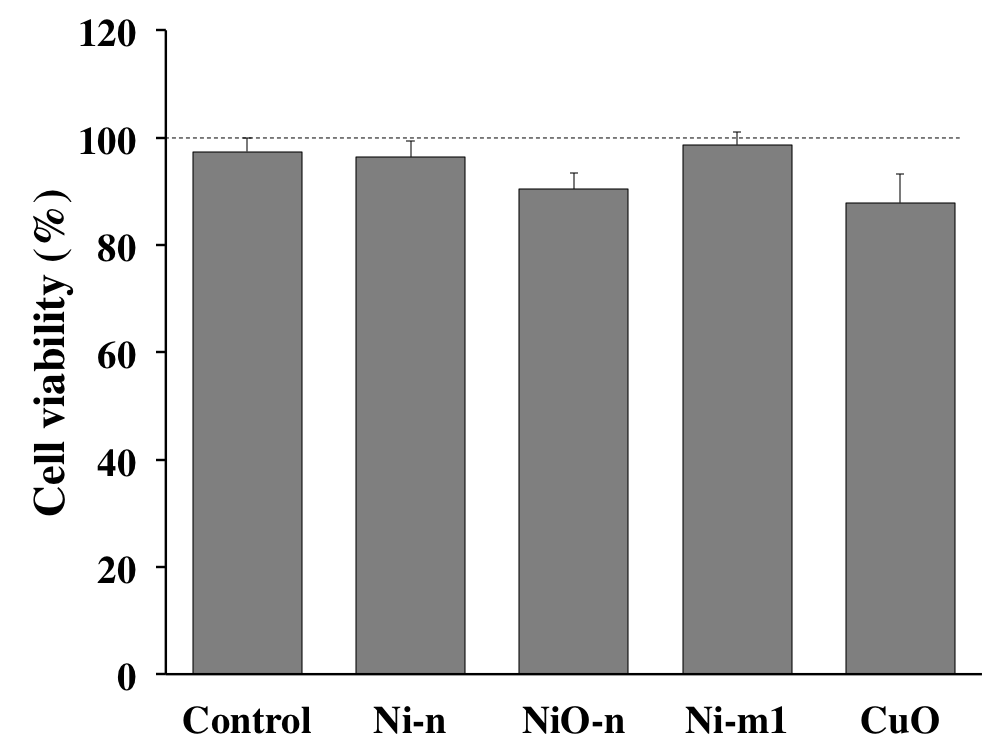

Supplement: S3 Fig — Cultured A549 cells after 4 h exposure to particle dispersions of Ni metal (Ni-n, Ni-m1, Ni-m2) and Ni oxide (NiO-n) of a total Ni concentration of 20 μg cm-2. Cells exposed to CuO-nanoparticle dispersion (20 μg cm-2) were used as positive control. Each bar represents the mean value of three independent experiments. (TIF) [file pone.0159684.s003.tif]

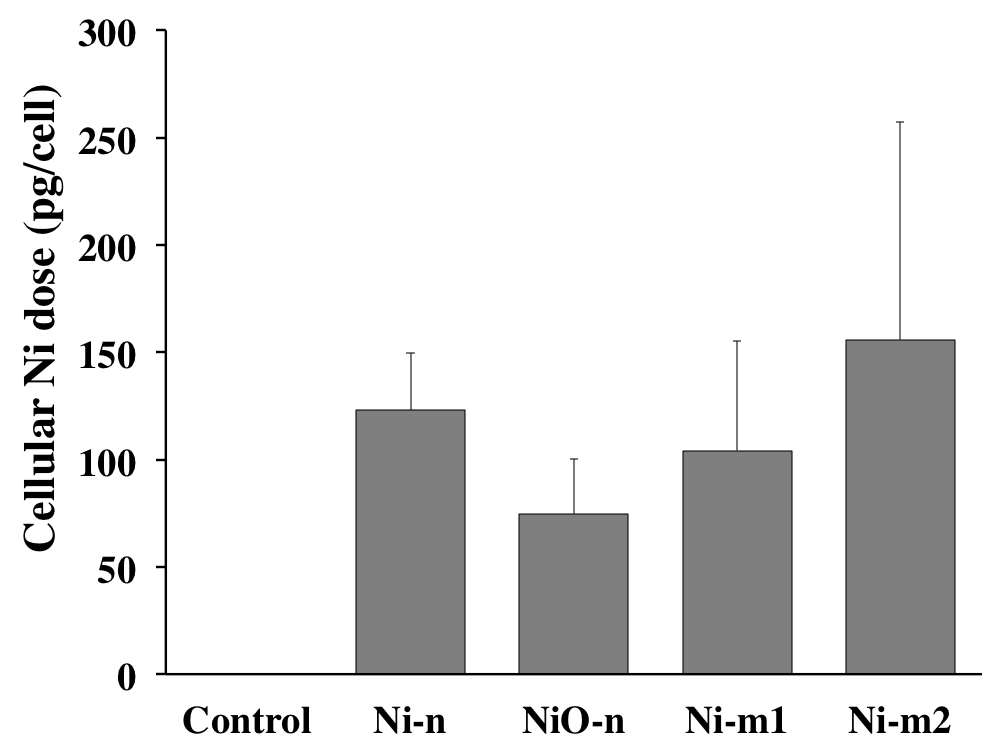

Supplement: S4 Fig — Cellular dose presented as the amount of Ni/cell. A549 cells after 4 h exposure to Ni metal (Ni-n, Ni-m1, Ni-m2) and Ni oxide (NiO-n) particle dispersions at total Ni concentration of 20 μg cm-2. Each bar represents the mean value of three independent experiments. (TIF) [file pone.0159684.s004.tif]

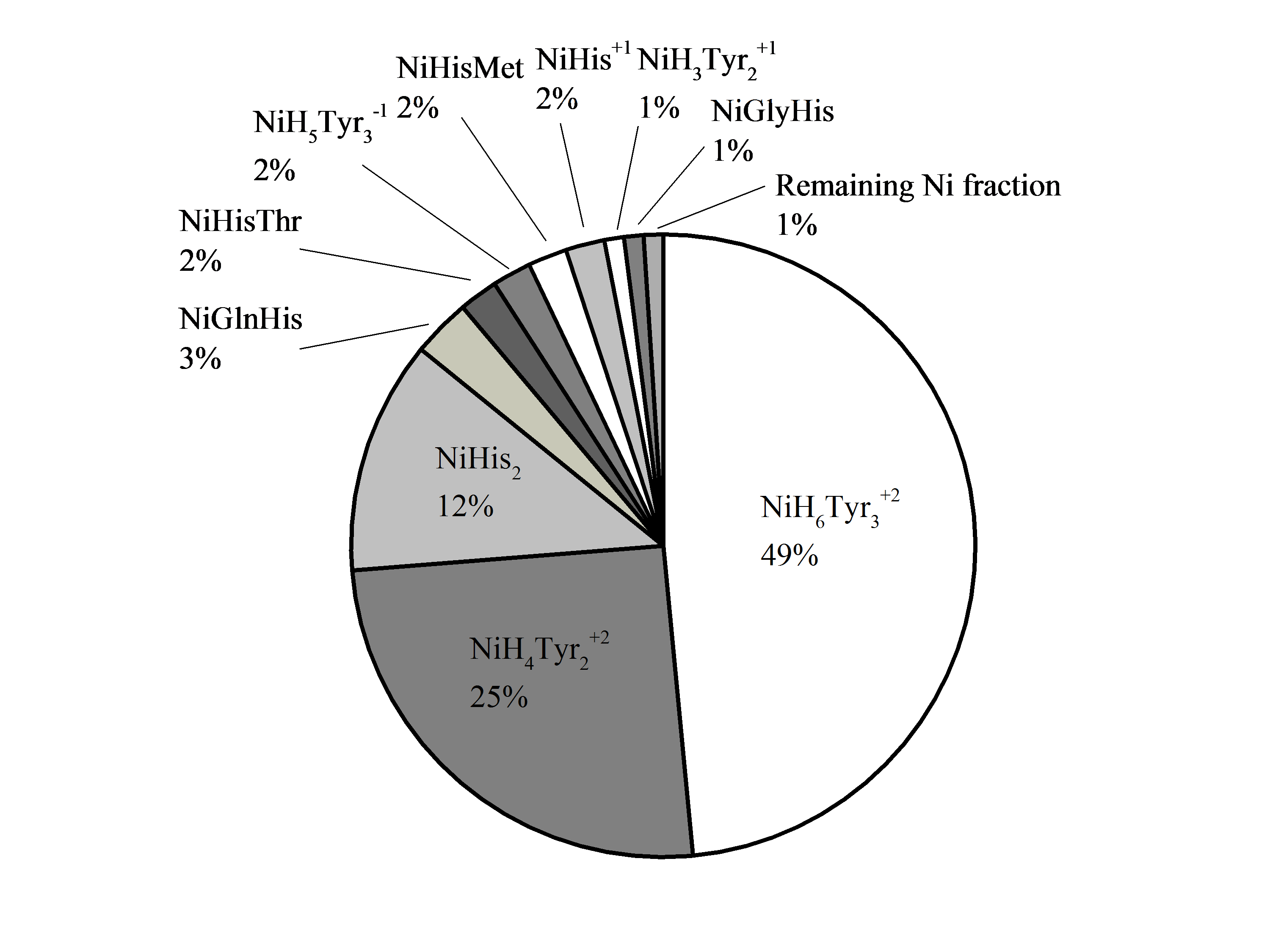

Supplement: S5 Fig — JESS solution speciation predictions of Ni in cell medium (DMEM; 10 μg mL-1). Abbreviations: Tyr = Tyrosine, His = Histidine, Gln = Glutamine, Thr = Threonine, Met = Methionine. (TIFF) [file pone.0159684.s005.tiff]
