# Supplementary material for: Nickel Release, ROS Generation and Toxicity of Ni and NiO Micro- and Nanoparticles
Source: PLoS One. 2016 Jul 19;11(7):e0159684. doi: 10.1371/journal.pone.0159684 (PMC4951072; doi:10.1371/journal.pone.0159684)
Supplement: S1 Table — The concentration of each compound that was contained in ALF is listed in this table. (DOCX) [file pone.0159684.s006.docx]

| Compound | Concentration [g L^-1^] |
| --- | --- |
| MgCl_2_ | 0.0497 |
| NaCl | 3.210 |
| Na_2_HPO_4_ | 0.071 |
| Na_2_SO_4_ | 0.039 |
| CaCl_2_·2H_2_O | 0.128 |
| NaOH | 6.0 |
| Citric acid | 20.8 |
| Glycine | 0.059 |
| C_6_H_5_Na_3_O_7_·2H_2_O | 0.077 |
| C_4_H_4_O_6_Na_2_·2H_2_O | 0.09 |
| C_3_H_5_NaO_3_ | 0.085 |
| C_3_H_3_O_3_Na | 0.086 |
