# Supplementary material for: Nickel Release, ROS Generation and Toxicity of Ni and NiO Micro- and Nanoparticles
Source: PLoS One. 2016 Jul 19;11(7):e0159684. doi: 10.1371/journal.pone.0159684 (PMC4951072; doi:10.1371/journal.pone.0159684)
Supplement: S2 Table — The concentration of each compound in DMEM that was used in the JESS solution speciation predictions for Ni. (DOCX) [file pone.0159684.s007.docx]

| Compound | Concentration [mM] |
| --- | --- |
| Fe^3+^ | 0.0284 |
| Cr^3+^ | 0.00025 |
| Ni^2+^ | 0.00067 |
| Mn^2+^ | 0.00038 |
| Glycine | 0.4 |
| L-Arginine hydrochloride | 0.4 |
| L-Cystine 2HCl (S-S) | 0.2 |
| L-Glutamine | 4 |
| L-Histidine hydrochloride-H_2_O | 0.2 |
| L-Isoleucine | 0.8 |
| L-Leucine | 0.8 |
| L-Lysine hydrochloride | 0.8 |
| L-Methionine | 0.2 |
| L-Phenylalanine | 0.4 |
| L-Serine | 0.4 |
| L-Threonine | 0.8 |
| L-Tryptophan | 0.08 |
| L-Tyrosine disodium salt dihydrate | 0.4 |
| L-Valine | 0.8 |
| Folic Acid | 0.009 |
| Pyridoxine hydrochloride | 0.02 |
| Riboflavin | 0.001 |
| Thiamine hydrochloride | 0.01 |
| Calcium Chloride (CaCl_2_) (anhyd.) | 1.8 |
| Ferric Nitrate (Fe(NO_3_)_3_"9H_2_O) | 0.0003 |
| Magnesium Sulfate (MgSO_4_) | 0.8 |
| Potassium Chloride (KCl) | 5.3 |
| Sodium Bicarbonate (NaHCO_3_) | 44 |
| Sodium Chloride (NaCl) | 110 |
| Sodium Phosphate monobasic (NaH_2_PO_4_-H_2_O) | 0.9 |
| D-Glucose (Dextrose) | 25 |
| Phenol Red | 0.04 |
