# Supplementary material for: Nickel Release, ROS Generation and Toxicity of Ni and NiO Micro- and Nanoparticles
Source: PLoS One. 2016 Jul 19;11(7):e0159684. doi: 10.1371/journal.pone.0159684 (PMC4951072; doi:10.1371/journal.pone.0159684)
Supplement: S1 File — (DOCX) [file pone.0159684.s008.docx]

**Cell viability upon released nickel exposure.** These fractions were prepared by incubating particle dispersions (total nickel concentrations of 0.2, 2, 10, 20, 40 and 80 μg mL^-1^ in DMEM^+^) in cell-free conditions for 24 h (37 **°**C, 5 % CO_2_). The particles were separated by centrifugation (20800 rcf, 10 min, 20 °C) and the supernatants (containing the released nickel fraction) were diluted with a factor of 2 using fresh DMEM^+^. Cells were then exposed to the released fractions corresponding to nickel concentrations of 0.1, 1, 5, 10, 20 and 40 μg cm^-2^, and analysed with the Alamar Blue assay.
