# Supplementary material for: Nickel Release, ROS Generation and Toxicity of Ni and NiO Micro- and Nanoparticles
Source: PLoS One. 2016 Jul 19;11(7):e0159684. doi: 10.1371/journal.pone.0159684 (PMC4951072; doi:10.1371/journal.pone.0159684)
Supplement: S2 File — (DOCX) [file pone.0159684.s009.docx]

**Chemical equilibrium modeling.** The Joint Expert Speciation Software (JESS, version 8.3 (1)) was used for chemical equilibrium calculations to make predictions on the speciation of Ni in cell medium. The redox potential of DMEM (360 mV vs. Ag/AgCl) was measured with an Inlab redox electrode (Mettler Toledo, Sweden), and used in the JESS modeling. The redox electrode was calibrated with standards from Thermo Scientific, Sweden (Lot # 967961). Same Ni concentration (10 µg mL^-1^) was used as in the release experiments. The temperature was set to 37 °C. A few components of DMEM (choline, pantothenate, niacinamide, inositol) were not available in the JESS database, and hence not included in the calculations. The solution components of DMEM used in the modeling are compiled in S2 Table**.**

According to the JESS modeling predictions of Ni speciation, essentially all Ni (100 %) forms complexes with amino acids in cell medium (S5 Fig). The fraction denoted “remaining Ni fraction” in S5 Fig, consists of other, non-specified amino acid-Ni -complexes. JESS is not capable of modeling proteins, and hence DMEM was used as a proxy for speciation in DMEM^+^. Ni binds readily to proteins (*e.g.* albumin), and therefore Ni is expected to have a protein-bound fraction in DMEM^+^ in addition to a similar amino acid -bound fraction as shown in S5 Fig. Therefore, according to these simulations, the released Ni in cell medium would not be present as labile or aquatic Ni complexes, which may affect the cellular uptake of the released Ni species (2-4).

References

[1] May PM. JESS at thirty: Strengths, weaknesses and future needs in the modelling of chemical speciation. Appl Geochem 2015 4;55:3-16.

[2] Glennon JD, Sarkar B. Nickel(II) transport in human blood serum. Studies of nickel(II) binding to human albumin and to native-sequence peptide, and ternary-complex formation with l-histidine. Biochem J 1982 04/01;203(1):15-23.

[3] Horie M, Nishio K, Fujita K, Endoh S, Miyauchi A, Saito Y, et al. Protein adsorption of ultrafine metal oxide and its influence on cytotoxicity toward cultured cells. Chem Res Toxicol 2009 03/16; 2015/02;22(3):543-553.

[4] Abbracchio MP, Evans RM, Heck JD, Cantoni O, Costa M. The regulation of ionic nickel uptake and cytotoxicity by specific amino acids and serum components. Biological Trace Element Research 1982;4(4):289-301.
